# Isolated Pulmonary Valve Endocarditis

**DOI:** 10.7759/cureus.8650

**Published:** 2020-06-16

**Authors:** Sudeep Acharya, Shamsuddin Anwar, Michael Iannuzzi, Viswajit Anugu, Foad Ghavami

**Affiliations:** 1 Internal Medicine, Staten Island University Hospital, Northwell Health, Staten Island, USA

**Keywords:** pulmonary valve, bacteremia, clinical infectious medicine, pulmonary disease, endocarditis

## Abstract

Infective endocarditis involving the right side of the heart is typically associated with IV drug abuse and chronic indwelling catheters which commonly involve the tricuspid valve. Isolated pulmonary valve endocarditis (PVE) is a rare clinical entity. We report a rare case of a young woman with a history of end-stage renal disease (ESRD) on hemodialysis through tunneled catheter presenting with persistent coagulase-negative staphylococcus (CoNS) epidermidis bacteremia despite being on appropriate treatment with IV vancomycin for two weeks. Because of the persistent bacteremia, a transesophageal echocardiogram was performed and it revealed a thickened pulmonary valve with 1.8 cm vegetation in the left posterior cusp. She was successfully treated with IV daptomycin course for a total of six weeks. The recommended management for PVE is usually medical treatment with IV antibiotics gauged according to sensitivities of the cultures. Our article highlights the fact that the decision to manage it medically versus surgically can propose a challenge as the guidelines are not very robust.

## Introduction

Isolated pulmonary valve endocarditis (PVE) is an extremely rare condition. We present a patient with PVE caused by coagulase-negative staphylococcus (CoNS) epidermidis that was successfully treated with IV antibiotics. The recommended management for PVE is usually medical treatment with IV antibiotics gauged according to sensitivities of the cultures. The surgical intervention in right-sided native valve endocarditis can be considered in recurrent septic pulmonary embolic, persistent bacteremia with highly resistant organisms and vegetations ≥ 20 mm in diameter. Our patient was managed as per the current guidelines with IV antibiotics [1]. Isolated PVE is a rarely reported entity, so this article highlights the significance of its appropriate treatment.

## Case presentation

A 30-year-old woman with end-stage renal disease (ESRD) due to polycystic kidney disease on hemodialysis through a tunneled catheter was referred by her nephrologist for persistent CoNS bacteremia, low-grade fever, malaise, and myalgia for more than two weeks. Her first outpatient blood cultures grew oxacillin resistant staphylococcus epidermis (sensitivities are shown in Table 1) and the patient was appropriately started on vancomycin (renally dosed). As the patient continued to have symptoms and blood cultures collected at different occasions were persistently positive for the same organism despite being on vancomycin, it prompted the outpatient nephrologist to refer the patient for hospitalization and further evaluation.  

**Table 1:** Sensitivities. Blood cultures, R=resistant to the antibiotic, S=sensitive to the antibiotic.

Antibiotics	Sensitivities (mic)
Ampicillin/Sulbactam	Resistant (<=8/4)
Cefazolin	Resistant (<=4)
Clindamycin	Sensitive (0.5)
Erythromycin	Resistant (>4)
Gentamicin	Sensitive (<=1)
Oxacillin	Resistant (>2)
Penicillin	Resistant (>8)
Rifampin	Sensitive (<=1)
Tetra/Doxy	Sensitive (2)
Trimethoprim/Sulfamethoxazole	Sensitive (<=0.5/9.5)
Vancomycin	Sensitive (2)

**Figure 1:**
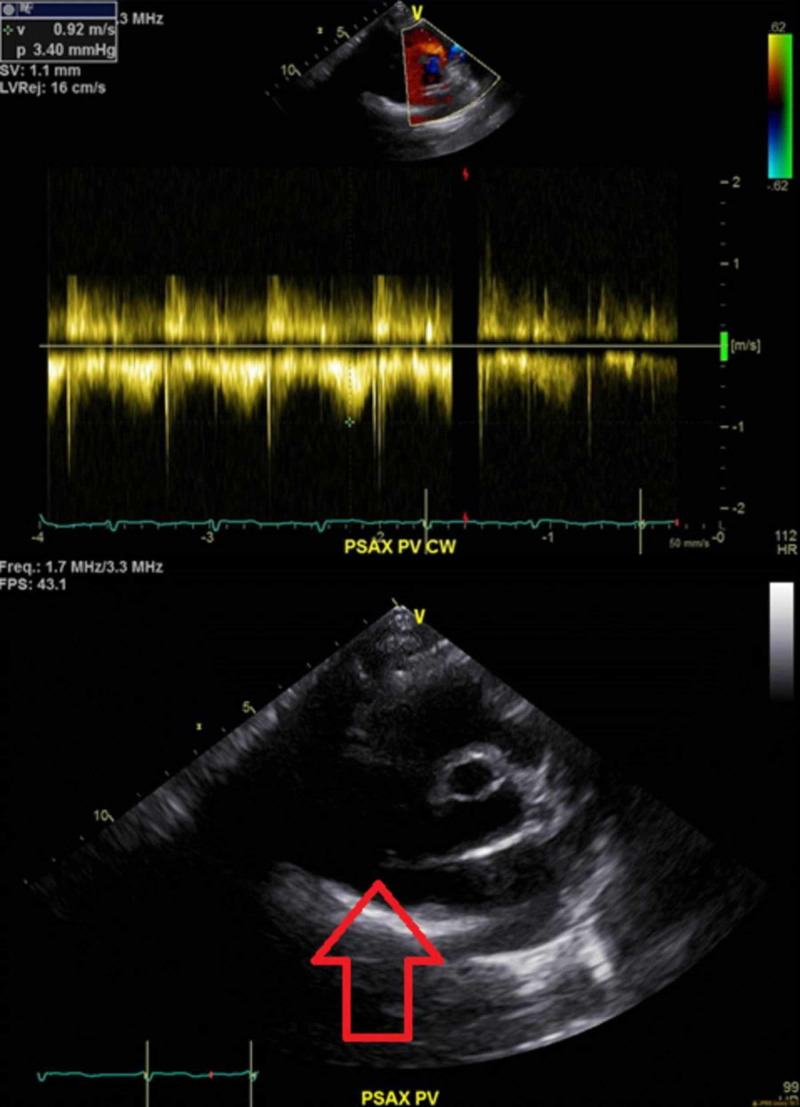
TTE. No evidence of vegetations on the valves. TTE, transthoracic echocardiogram

The patient was admitted to the hospital with a diagnosis of persistent bacteremia from the possible source being the tunneled hemodialysis catheter. She was started on IV daptomycin as she failed to respond to vancomycin. A transthoracic echocardiogram (TTE) was obtained upon admission to screen for endocarditis (Figure 1 ) which did not show any vegetations and ruled out any underlying valvular diseases including pulmonary regurgitation.

**Figure 2:**
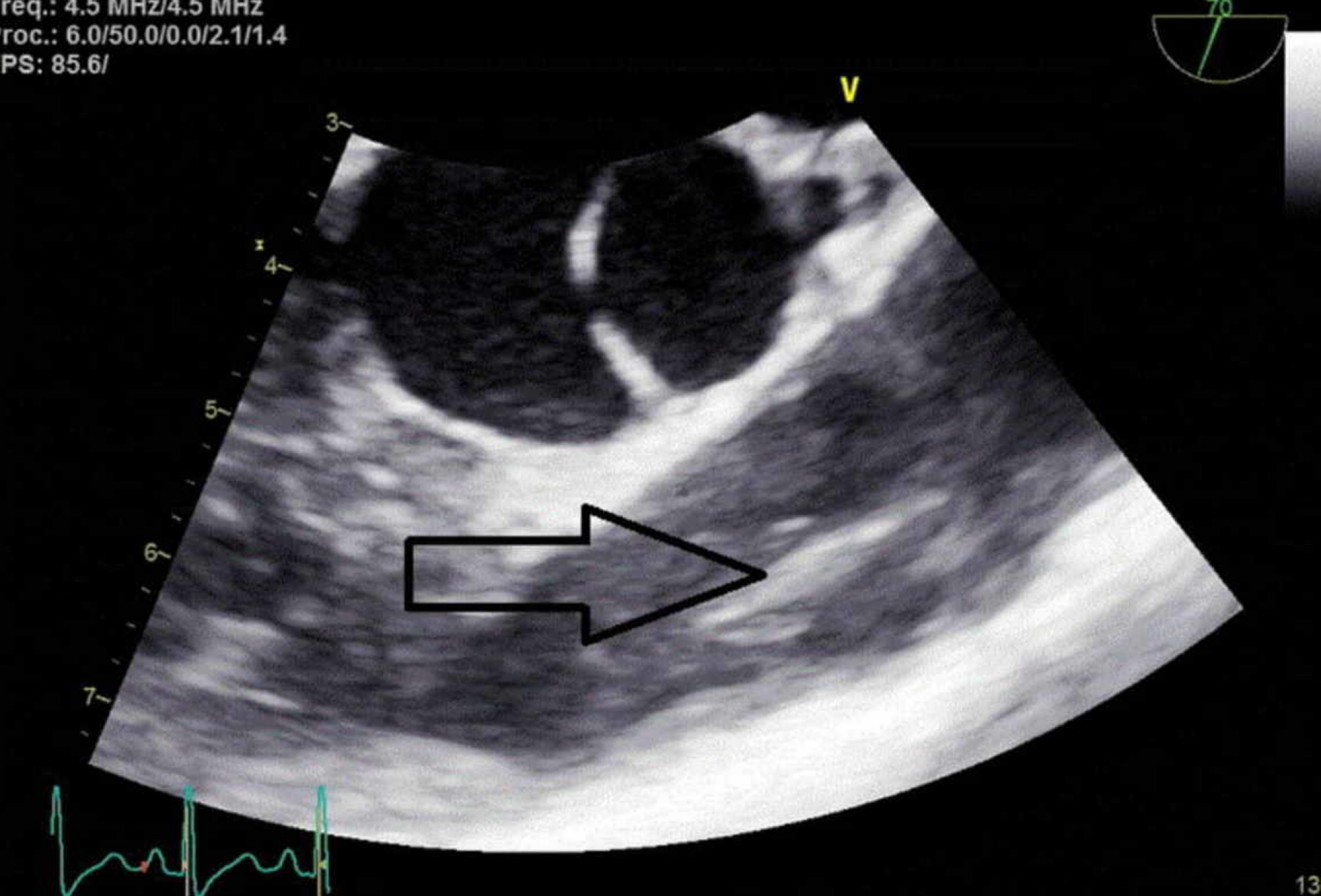
Transesophageal echocardiogram. Pulmonary valve vegetation as shown by the arrowhead.

Given a high index of suspicion for endocarditis, a transesophageal echocardiogram was performed which revealed a thickened pulmonary valve with elongated, mobile, 1.8 cm vegetation on the left leaflet of the pulmonary valve without evidence of valvular regurgitation (Figure 2). The bacteremia resolved with IV daptomycin treatment and the patient underwent replacement of a tunneled dialysis catheter.

The patient was also evaluated by cardiothoracic surgery; however, surgical intervention was not recommended in view of clinical improvement and no significant damage to the valve.

**Figure 3:**
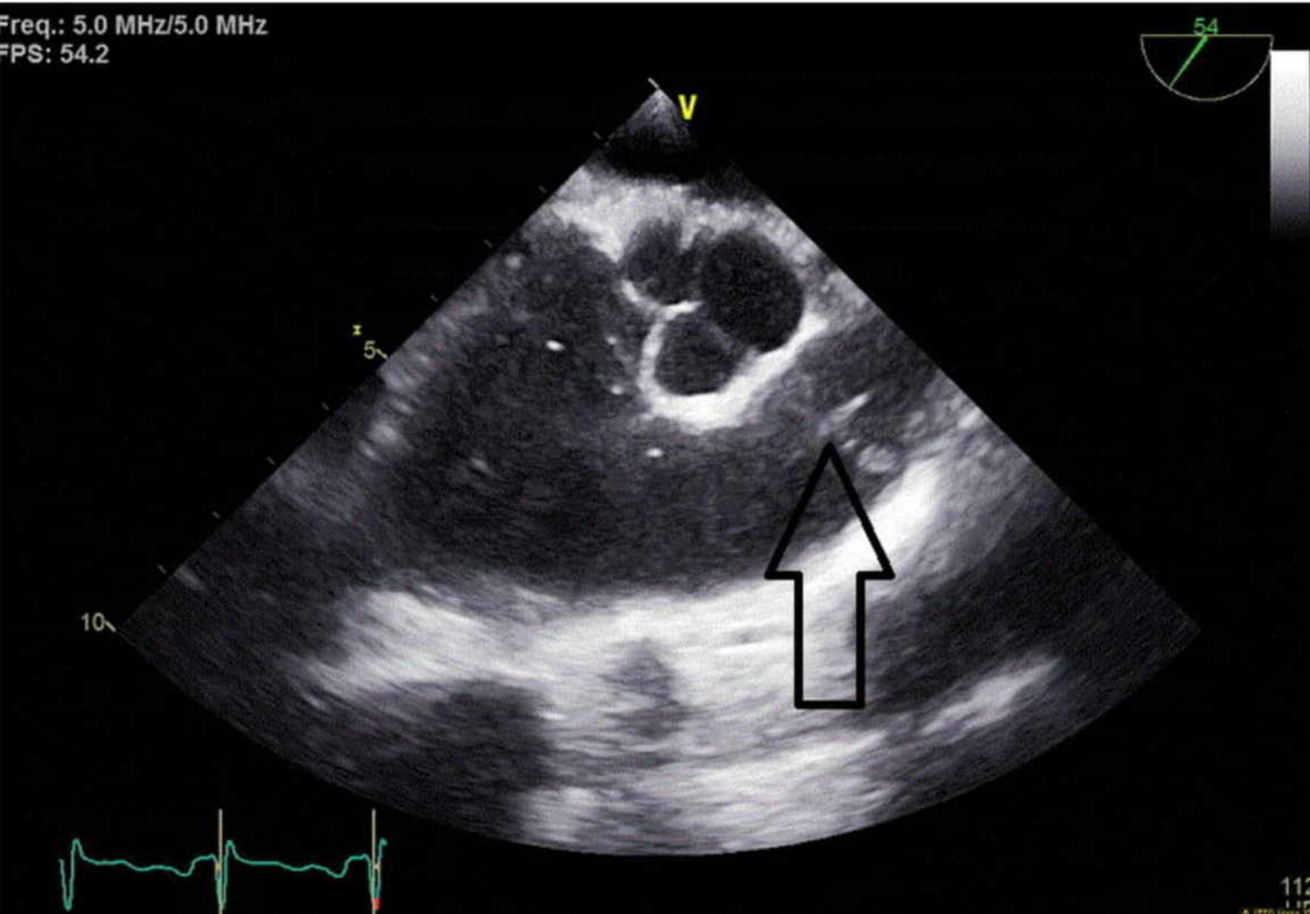
Transesophageal echocardiogram. Resolution of vegetation after intravenous therapy.

A repeat TEE was performed after the completion of six weeks of daptomycin therapy which showed complete resolution of the vegetation with preserved valve function (Figure 3).

## Discussion

Isolated PVE is an extremely rare entity to be described in the medical literature which can present with nonspecific signs and symptoms such as fever, pleuritic chest pain, hemoptysis, and delayed onset diastolic murmur [2-4]. The common precipitating factor for PVE includes IV drug use, immunosuppression, valvular replacement, congenital heart diseases, alcoholism, and catheter-related infections as suggested in our case described [2, 4-5]. IV drug use is the most common (90%) cause of infective endocarditis on the right side of the heart and majorly involves the tricuspid valve (>90%) [6]. Given the low incidence of PVE (1.5%-2%), it can pose a challenge for physicians for prompt diagnosis hence risking timely management of the infection [7]. 

In general, TTE is usually performed as the first diagnostic test in patients suspected of infective endocarditis. Even though the sensitivity of TTE is high (40%-63%), normal valvular imaging and function do not exclude the suspicion of endocarditis. In this situation, TEE is performed which has higher sensitivity (90%-100%) in detecting vegetations and valvular abscesses [8-9]. As discussed above in our case, the initial TTE failed to reveal any pulmonary valve abnormality even though anatomically speaking the pulmonary valve is closer to the chest wall. Later, TEE was able to detect 1.8 cm mobile mass on the pulmonary valve confirming the diagnosis of infective endocarditis. 

Several microorganisms have been identified for causing infective endocarditis in pulmonary valves which includes Staphylococcus species (>50%), Streptococcus species (>10%), Enterococcus species (9%), and fungal organism (>4%). Infections with CoNS account for roughly 2% of the PVE among Staphylococcus species. As the incidence of PVE with CoNS is so low, this article is unique in describing an interesting case of Staphylococcus epidermidis PVE with complete resolution [10-11].

Pulmonary valve endocarditis usually follows a benign course and responds appropriately to medical management with IV antibiotics along with supportive care [12]. The role of surgical intervention has been discussed in persistent bacteremia especially with resistant organisms, complications such as abscess formation, and recurrent septic pulmonary embolism. Hemodynamic un-stability, vegetations >20 mm, and Staphylococcus infection are also considered indications for surgical consultation in right-sided endocarditis [13-17]. As described above in our patient’s clinical course, surgical intervention was deferred because she responded well and appropriately with medical management. Nevertheless, it is important to understand that surgical consultation was appropriate from the beginning of the clinical course, should there be any evidence of clinical deterioration, lack of response to antibiotic therapy, or superimposed impressive pulmonary valve stenosis or insufficiency. 

It was interesting to note that this was the second time our patient had bacteremia with Staphylococcus epidermidis. The previous episode of bacteremia was reported in 2016 and the patient responded well with IV antibiotics and the tunneled catheter was replaced (sensitivities are shown in Table 2). During current hospitalization, it was interesting to note that even though the blood cultures for Staphylococcus epidermis bacteria were sensitive to vancomycin (MIC 2), the blood infection failed to respond to vancomycin. The patient was initiated on daptomycin with a dosing of 6 mg/kg posthemodialysis which resulted in clearing off the bacteremia. She was continued on this antibiotic regimen for a total of six weeks with close monitoring of creatine phosphokinase. There has been limited data in the usage and efficacy of daptomycin against CoNS infections though daptomycin has been shown to have good in vitro bactericidal activity in oxacillin resistant CoNS infections [18-19]. Our clinical scenario provides substantial evidence that daptomycin can be used as an alternative for oxacillin resistant CoNS infections with good results. 

 

**Table 2:** Sensitivities. Previous blood cultures in 2016, R=resistant to the antibiotic, S=sensitive to the antibiotic.

Antibiotics	Sensitivities (mic)
Ciprofloxacin	Sensitive (<=1)
Levofloxacin	Sensitive (<=1)
Clindamycin	Resistant (>4)
Erythromycin	Resistant (>4)
Gentamicin	Intermediate (8)
Oxacillin	Sensitive (<=0.25)
Penicillin	Resistant (2)
Rifampin	Sensitive (<=1)
Tetra/Doxy	Sensitive (<=4)
Trimethoprim/Sulfamethoxazole	Sensitive (<=0.5/9.5)
Vancomycin	Sensitive (4)

## Conclusions

This article summarizes a unique case of isolated PVE with CoNS from the tunneled hemodialysis catheter. As this condition is extremely rare and diagnosis is often challenging, a high index of suspicion should be maintained when evaluating high-risk patients. It is important to keep in mind that endocarditis can often be missed on a simple TTE and it is almost mandatory to get a transesophageal echocardiogram to visualize all the cardiac structures appropriately. Treating endocarditis involves multi-specialties of medicine and surgery which is duly suggested for its proper management. 

## References

[1] Baddour LM, Wilson WR, Bayer AS (2015). Infective endocarditis in adults: diagnosis, antimicrobial therapy, and management of complications: a scientific statement for healthcare professionals from the American Heart Association. Circulation.

[2] Ranjith MP, Rajesh KF, Rajesh G, Haridasan V, Bastian C, Sajeev CG, Krishnan MN (2013). Isolated pulmonary valve endocarditis: a case report and review of literature. J Cardiol Cases.

[3] Seraj SM, Gill E, Sekhon S (2017). Isolated pulmonary valve endocarditis: truth or myth?. J Community Hosp Intern Med Perspect.

[4] Swaminath D, Yaqub Y, Narayanan R, Paone RF, Nugent K, Arvandi A (2013). Isolated pulmonary valve endocarditis complicated with septic emboli to the lung causing pneumothorax, pneumonia, and sepsis in an intravenous drug abuser. J Investig Med High Impact Case Rep.

[5] Kamaraju S, Nelson K, Williams DN, Ayenew W, Modi KS (1999). Staphylococcus lugdunensis pulmonary valve endocarditis in a patient on chronic hemodialysis. Am J Nephrol.

[6] Bentata Y, Haddiya I, Ismailli N, El Ouafi N, Benzirar A, El Mahi O, Azzouzi A (2016). Infective endocarditis in chronic hemodialysis: a transition from left heart to right heart. Saudi J Kidney Dis Transpl.

[7] Ramadan FB, Beanlands DS, Burwash IG (2000). Isolated pulmonic valve endocarditis in healthy hearts: a case report and review of the literature. Can J Cardiol.

[8] Daniel WG, Mügge A, Martin RP (1991). Improvement in the diagnosis of abscesses associated with endocarditis by transesophageal echocardiography. N Engl J Med.

[9] Samaroo-Campbell J, Hashmi A, Thawani R, Moskovits M, Zadushlivy D, Kamholz SL (2019). Isolated pulmonic valve endocarditis. Am J Case Rep.

[10] Yuan S-M (2014). Right-sided infective endocarditis: recent epidemiologic changes. Int J Clin Exp Med.

[11] Chang C-H, Huang M-M, Yeih D-F, Lu K-C, Hou Y-C (2017). A chronic hemodialysis patient with isolated pulmonary valve infective endocarditis caused by non-albicans Candida: a rare case and literature review. BMC Nephrol.

[12] Saleem M, Ahmed F, Patel K, Munir MB, Ghaffar YA, Mujahid H, Balla S (2019). Isolated pulmonic valve endocarditis: case report and review of existing literature on diagnosis and therapy. Case.

[13] Martín-Dávila P, Navas E, Fortún J (2005). Analysis of mortality and risk factors associated with native valve endocarditis in drug users: the importance of vegetation size. Am Heart J.

[14] Vrettos A, Mota P, Nash J, Thorp I, Baghai M, Baghai M (2017). Pneumococcal pulmonary valve endocarditis. Echo Res Pract.

[15] Lyle M, Espinosa R (2018). Pulmonary valve endocarditis. J Am Coll Cardiol.

[16] Kurnicka K, Nowakowski P, Pruszczyk P (2017). A rare case of isolated streptococcal pulmonary valve endocarditis diagnosed with repeated echocardiography. Pol Arch Intern Med.

[17] Akinosoglou K, Apostolakis E, Koutsogiannis N, Leivaditis V, Gogos CA (2012). Right-sided infective endocarditis: surgical management. Eur J Cardiothorac Surg.

[18] Fowler VG Jr, Boucher HW, Corey GR (2006). Daptomycin versus standard therapy for bacteremia and endocarditis caused by Staphylococcus aureus. N Engl J Med.

[19] Guleri A, Utili R, Dohmen P, Petrosillo N, Piper C, Pathan R, Hamed K (2015). Daptomycin for the treatment of infective endocarditis: results from European Cubicin(®) Outcomes Registry and Experience (EU-CORE). Infect Dis Ther.

